# The extraterrestrial search for our own chemical origins

**DOI:** 10.1038/s41467-023-41009-y

**Published:** 2023-09-25

## Abstract

The study of Prebiotic Chemistry, and the closely related study of Astrobiology, is ultimately the study of our own point(s) of origin. Aiming to answer the questions of how, when, and where did the building blocks of life—i.e. biologically relevant organic molecules—form? With the imminent analysis of samples successfully returned from the near-Earth asteroid Bennu, and continuing discoveries from the Ryugu asteroid samples, the answers to some of these questions may be in sight.

**Figure:**
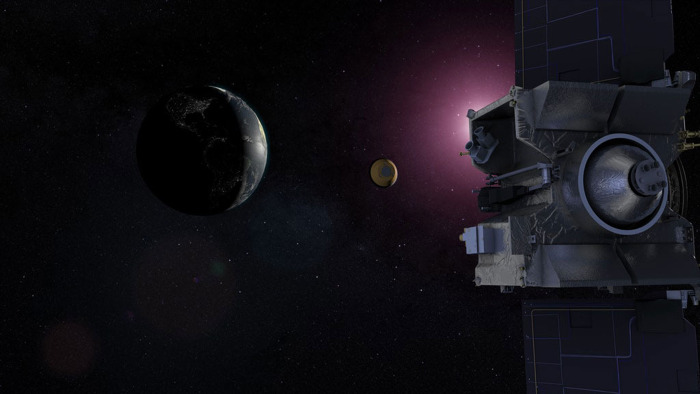
An artist’s depiction of the sample return capsule ejecting from the OSIRIS-REX spacecraft towards Earth. Credit: NASA’s Goddard Space Flight Center Conceptual Image Lab, still from animation https://gms.gsfc.nasa.gov/20257#media_group_15943

To answer these fundamental questions, we need ancient, biologically relevant, organic molecules that have been preserved to the present day. Unfortunately, owing to the continued recycling of crust by subduction, it is rare to find rock dating to before life’s origins on Earth. Where the most ancient (Hadean) terrestrial material does survive it is often only found as isolated crystals^1^, themselves modified by the extreme conditions of continental formation and destruction. The easily adaptable nature of organic molecules, which makes them a useful base for biology, ensures that their preservation is nearly impossible on Earth.

Even where signatures of prebiotic chemistry may persist^2^, it is hard—if not impossible—to identify them due to the ubiquity of biology for much of Earth’s history. Life is so pervasive that it alters chemical processes even deep within the Earth’s crust^3^. Because of this, to identify processes with neither biotic precursors nor biological modifications, we must look outside of the terrestrial sphere.

Asteroids and comets are rich in organic matter, the most primordial of which may have formed in the interstellar medium prior to the birth of the solar system. It has long been suggested that the delivery of organic carbon by meteorites may have been necessary for the development of prebiotic chemistry on Earth^4,5^. However, what biologically relevant molecules had already formed extraterrestrially is a continuing topic of research.

By studying the organic content of meteorites^6^, insights have been made into the nature of prebiotic molecules which may have been delivered to the early Earth, potentially kickstarting the chain of events leading to life. However, it is difficult to conclusively constrain the primary extraterrestrial nature of many of the organic molecules detected in meteorites. Many well-studied meteorites sat on the surface of Earth for years before being found, being exposed to weathering, aqueous alteration, and providing plenty of time for microbial life to colonise and contaminate. Even falls tracked and collected within days do not escape contamination, as carbon-rich meteorites are rapidly occupied by terrestrial microbes^7^.

Many of the problems we associate with billions of years of active geology and biology on Earth can be overcome by departing this world. Remotely exploring other rocky bodies, elsewhere in the solar system, via robotic orbiters and landed missions makes it possible to analyse pristine extraterrestrial samples in situ. These samples may evidence true prebiotic organic mechanisms, shedding light on processes which also occurred on the earliest Earth, or even at the birth of the solar system.

However, in situ analysis brings with it its own suite of problems. Not all instruments can be miniaturised and flown, and it is necessary to sacrifice precision and detection limits for portability and energy efficiency. There are rarely enough resources (or physical space) for redundancy, thus multiple complementary techniques are unlikely to be selected. It is therefore difficult to work around unforeseen complications, which may arise from the operation of instruments in an ‘alien’ environment. For example, highly oxidising salts in martian soil have degraded extraterrestrial organic matter during thermal decomposition experiments, leading to ambiguous results^8^. These difficulties will be compounded on future missions that operate further out and in even more complex environments. For example, Dragonfly, to be launched later this decade, will land on Saturn’s organic-rich moon Titan^9^. With Titan’s methane seas and hazes of high molecular weight aerosols^9^, it will be the complexity, rather than scarcity, of organic chemistry that may present difficulties.

Therefore, if we want to have pristine, unaltered samples free from terrestrial contaminants, and the high precision and multi-technique reliability of identification of an Earth-based lab (i.e. the best of both worlds), we need sample return missions. So far, samples have been brought back from our moon^10^ by the Apollo, Luna, and Chang’e-5 missions; the comet Wild 2/81P by Stardust; the near-Earth asteroids Ikotawa and Ryugu^11^ by Hayabusa 1 and 2; and now from Bennu by OSIRIS-REX. Robotic missions delivering samples collected from extraterrestrial environments to clean terrestrial laboratories may be technically demanding, but it allows pristine samples to be interrogated with the most advanced, cutting-edge analytical techniques. We see the fruits of this with the detection of biologically-relevant prebiotic organic compounds, such as nucleotides^12^, in minute concentrations in returned samples from the asteroid Ryugu. Carefully controlled sample handling chains minimise doubts about the extraterrestrial provenance of these biologically-relevant organic molecules.

“We will need more returned samples from a wider range of extraterrestrial environmental conditions to conclusively answer these fundamental issues of life’s existence.”

The samples collected during sample-return missions are incredibly precious. The difficulties and costs involved in these missions mean that only small volumes of material have so far been retrieved. For example, just 5.5 g were returned from Ryugu. And, as many of the necessary analytical techniques are destructive, this finite supply must be carefully curated. These practical limitations of sample-return missions, alongside the fact that it is not yet technically possible to return samples from all extraterrestrial environments of interest, mean that we cannot rely on these missions alone. In situ robotic analysis, ground truthing in analogous Earth environments, model and lab experiments are all complementary and required to further our understanding. They may also aid in focussing costly return efforts on the most potentially lucrative samples and targeting analysis of them on the most pressing questions.

In *Nature Communications*, we present a collection of papers published over the last few years, highlighting recent developments in all of these methods of investigation. And yet, there remain many questions to answer. The next stage of robotic exploration of the solar system may answer some of these questions, but it is clear that we will need more returned samples, from a wider range of extraterrestrial environmental conditions, to conclusively understand the fundamental origins of life. However, it is certain that with the ongoing analysis of samples from Ryugu, the imminent discoveries from samples of Bennu, the planned return of martian samples in the next decade^13^, and more speculative sample return concepts to the outer solar system being developed^14^, this is an exciting time in the study of prebiotic chemistry and astrobiology.
